# Functional seizures and their mimics: a retrospective service review of cases from a tertiary video telemetry database

**DOI:** 10.1136/bmjno-2024-000738

**Published:** 2024-08-06

**Authors:** Peter Dudley, Jan Paul Marquez, Fiona Farrell, Jennifer Benson, Fergus Rugg-Gunn, Meneka K Sidhu, Suzanne O'Sullivan, Matthew Walker, Mahinda Yogarajah

**Affiliations:** 1 Department of Epilepsy, Chalfont Centre for Epilepsy, Chalfont St Peter SL9 0RJ, UK; 2 Department of Clinical & Experimental Epilepsy, UCL Queen Square Institute of Neurology, London WC1N 3BG, UK; 3 NIHR University College London Hospitals Biomedical Research Centre, University College London, London, UK

**Keywords:** FUNCTIONAL NEUROLOGICAL DISORDER, EPILEPSY, EEG

## Abstract

**ABSTRACT:**

**Objective:**

Identify the proportion of patients referred with putative functional seizures (FS) that were subsequently re-diagnosed as epileptic seizures (ES), or an alternative diagnosis, following video telemetry EEG (VTEEG). In addition, describe the characteristics of those seizures.

**Methods:**

The VTEEG reports from patients admitted to the Chalfont Centre for Epilepsy between 2019 and 2022 were reviewed. Pre-VTEEG and post-VTEEG diagnoses were compared to identify whether a diagnostic revision was made from suspected FS to ES or another diagnosis. Diagnostic revision cases were then grouped into cohorts with associated features and reviewed to characterise and describe FS mimics.

**Results:**

444 VTEEG reports where patients had habitual events were identified. 4.7% of patients were referred with FS and were subsequently diagnosed with ES or another diagnosis. In this group, several cohorts could be identified including frontal lobe epileptic seizures, ES with functional overlay, insular or temporal lobe epileptic seizures associated with autonomic or marked experiential peri-ictal symptoms, and individuals who had both ES and FS but whose ES were revealed on medication withdrawal.

**Conclusion:**

In patients referred to a tertiary epilepsy unit, a small minority of cases had seizures diagnosed as functional and reclassified as epileptic or an alternative diagnosis. It is clinically important to be aware of these FS mimics.

**WHAT IS ALREADY KNOWN ON THIS TOPIC**

- Patients with functional seizures commonly present to neurology services. Their diagnosis when based on history alone is often challenging, and video telemetry EEG (VTEEG) provides a gold-standard level of diagnosis. Misdiagnosis of functional seizures is uncommon but case reports have previously highlighted frontal lobe seizures, seizures associated with autoimmune encephalitis and paroxysmal movement disorders as functional seizure mimics.

**WHAT THIS STUDY ADDS**

- This study uses a VTEEG database to identify the proportion of patients who prior to VTEEG have been misdiagnosed with functional seizures. It also identifies the typical scenarios when misdiagnosis may occur.

**HOW THIS STUDY MIGHT AFFECT RESEARCH, PRACTICE OR POLICY**

- This study will help clinicians in the diagnosis of patients presenting with seizure-like episodes by highlighting those rare cases where a clinician may misdiagnose a patient with functional seizures.

## Introduction

 Functional seizures (FS) occur in 2 to 33 people per 100 000 and are commonly mistaken as epileptic seizures (ES).^1 2^ This results in unnecessary medication burden and an estimated total of up to £138 million per year in healthcare costs in England and Wales.^3^,^5^

Differentiating between ES and FS is not straightforward with the misdiagnosis of epilepsy varying between 2% and 71%, of which FS form a significant proportion.^6^ This is a consequence of varying degrees of clinical exposure to FS and the poor accuracy of witness accounts of seizures.^7 8^ It is further complicated as an epilepsy diagnosis is present in 22% of patients with FS and a concurrent FS diagnosis in approximately 12% of epileptics.^9^ Finally, overlapping semiological features in both types of seizures can make differentiation difficult, especially, by non-experts.^4^

The literature primarily focuses on the misdiagnosis of ES, with few studies outside of case reports published on the mimics of FS, in part because this is a less common occurrence.^46 10^,^15^ In this study, we aimed to identify and describe FS mimics referred to our centre and ascertain the proportion that had a diagnostic revision following video telemetry EEG (VTEEG). As far as the authors are aware, this is the first study analysing VTEEG reports and patient records to detect FS mimics.

## Methods

The study included patients admitted to the Chalfont Centre for Epilepsy between January 2019 and March 2022 that underwent VTEEG. The pre-VTEEG working diagnosis was based on the analysis of VTEEG reports which summarise the reasons for referral to the VT unit. Where this information was not present or unclear, information was taken from case notes, referral and pre-admission clinic letters, and a consensus agreement as to the working diagnosis was reached by several of the authors (PD, JPM and MY). The final pre-VTEEG working diagnosis categories used were epileptic, functional, unclear or both. Where a consensus agreement could not be reached regarding the pre-VTEEG working diagnosis, the category ‘unclear’ was used. Those that remained unclear were part of the study cohort for the purpose of the denominator value when calculating the rate of diagnostic change from functional to epileptic seizures. The post-VTEEG diagnosis was categorised based on the VTEEG findings of the habitual event as epileptic, functional or an alternative diagnosis. Cases where there was a diagnostic revision from suspected FS to ES, or an alternative paroxysmal cerebral dysfunction, were grouped and reviewed to identify the commonalities where a diagnostic revision in this direction might take place. In the majority of patients referred to the video telemetry unit at the Chalfont Centre for Epilepsy, there is a provisional pre-admission diagnosis that has been made by the referrer. Typically, the primary reason for referral is to confirm and reinforce the diagnosis.

VTEEGs that did not capture habitual events were excluded from the study and were not included in the denominator value when calculating the diagnostic revision rate. This study was approved and reviewed by the National Hospital for Neurology and Neurosurgery Research Audit and Clinical Governance Committee as a service evaluation.

## Results

**Figure 1:**
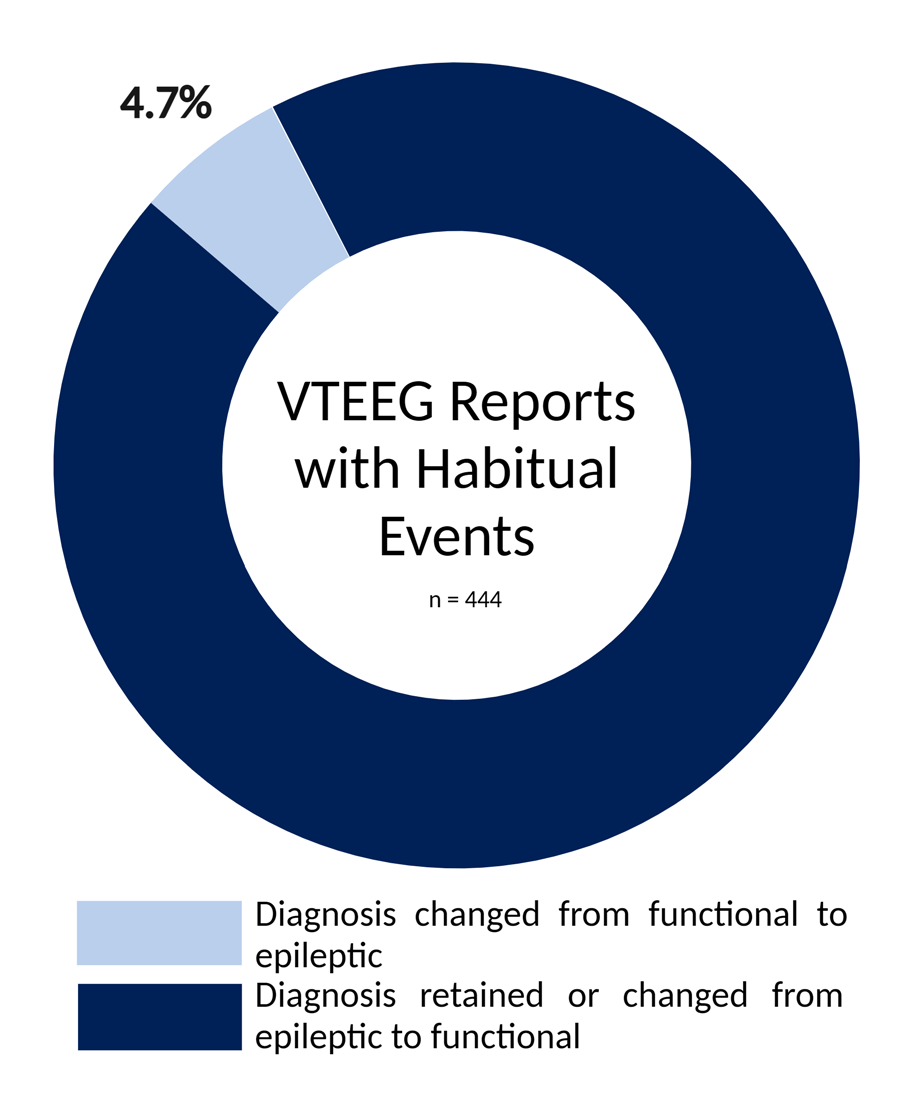
Percentage of patients that underwent a diagnostic revision from FS to ES/alternative paroxysmal cerebral pathology, in contrast to those whose diagnosis was unchanged or revised from ES to FS. FS, functional seizures; ES, epileptic seizures.

**Figure 2:**
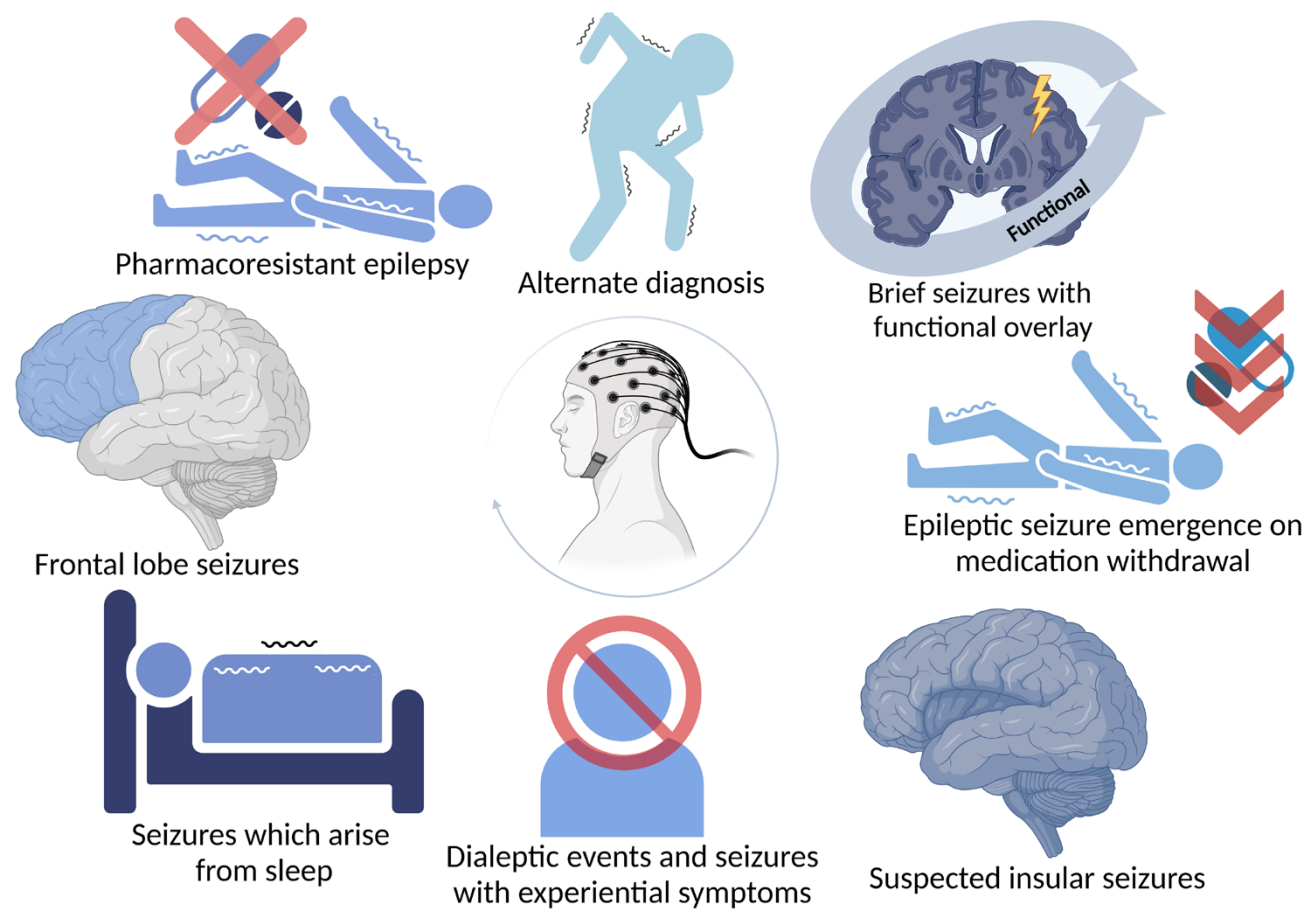
Identified functional seizure mimics.

Habitual events were recorded in 444 VTEEG reports, and the case notes of a total of 96 patients, where the outcome was that of an ES or both, were reviewed in order to ascertain the nature of the pre-EEG impression and whether a diagnostic revision had indeed occurred. In 21 (4.7%) cases, the diagnosis changed from FS to ES or an alternative paroxysmal cerebral dysfunction (figure 1). Several diagnostic revision groups were identified (figure 2) with a proportion of patients belonging to more than one category.

Of the 21 cases, one group identified were eight individuals (38%) with frontal lobe seizures. This group included stereotyped bizarre hypermotor seizures often occurring during sleep in the absence of ictal EEG changes. These cases had been misdiagnosed as FS due to a previous lack of ictal EEG correlates, as well as the bizarre nature of the seizure, which had been labelled as functional in nature. In 10 patients (48%), most had no ictal EEG abnormalities present, but stereotyped seizures arose during EEG-defined sleep. There was significant overlap between this cohort and those within the frontal lobe seizure category. In patients whose semiologies were not clearly attributable to frontal lobe epilepsy, one individual had multiple stereotypical events from sleep characterised by abrupt waking, confusion, difficulty in breathing with laryngeal symptoms, including a feeling of choking, and severe headache. These stereotypical events also occurred during wakefulness, excluding parasomnias. It was proposed that there was likely a focal epilepsy with some frontal lobe/insular features but not enough evidence for a definitive localisation. The episodes resolved with antiseizure medications (ASMs). Another patient with a predisposing liability for seizures had episodes of jerking of the left arm followed by jerking of all four limbs. These had been captured on a shorter previous study and had no ictal correlates, with the semiology of the generalised jerking in keeping with FS. However, on subsequent longer VTEEG, it was noted that the subtle initial jerking also appeared while the patient was in EEG-defined sleep, and that there was subsequent behavioural elaboration. It was therefore postulated that despite the absence of ictal correlates because the initial onset of the event arose from sleep, this suggested the aetiology was a focal epilepsy.

VTEEG also highlighted a group of 5 individuals (24%) with ES followed by a behavioural elaboration or functional overlay. These ES were brief, subtle and typically manifested with for example eyelid myoclonia, changes in facial expression, a brief pause in or loss of awareness or consciousness or head turning in the presence of EEG correlates. Their brevity and subtle nature meant witnesses had typically not noticed these features. The subsequent FS elaboration presented more obviously with features including irregular body jerks, head shaking, muddled speech and protracted periods of emotional upset. The elaborations typically followed the cessation of electrographic seizure activity. However, in one case, the unusual behaviour occurred during increased frequency of widespread polyspike waves of an absence seizure.

Another group of 4 cases (19%) highlighted the challenges in discerning whether periods of a dialeptic nature where an individual has a brief loss of pause in awareness or consciousness but without added signs were functional or epileptic from history alone.

Two cases (10%) had a historical but unsubstantiated diagnosis of epilepsy and had been referred because the current seizure burden was clearly functional in nature based on prior VTEEG recording of captured seizures and normal interictal findings. In both cases, it was clear from the documented available medical records that the patients had not had any documented seizures with clear epileptic features. In both cases, therefore, the pre-VTEEG diagnosis was thought to be one of FS, and the cases had been referred with this as the primary diagnosis with little credence given to the historical epilepsy diagnosis. Following ASM withdrawal, both subsequently experienced the emergence of new events, different in semiology to their FS, which were subsequently confirmed on VTEEG as epileptic, confirming a dual diagnosis.

In one case (5%), a diagnosis of FS was amended to insular epilepsy. Although this diagnosis had previously been made, it was revised to FS at a different centre due to the lack of ictal EEG correlates and poor response to ASMs. During our study, the stereotypy of the attacks, the associated bradycardia and successful response to a novel ASM implicated an epileptic aetiology.

In another case, a single individual (5%) was thought to have paroxysmal kinesigenic dyskinesia (PKD) and not FS as originally diagnosed. In support of PKD were the events dystonic nature and triggered by movement. The patient was commenced on carbamazepine and a paroxysmal central nervous system gene panel was sent. A conclusive outcome of this case is not available due to pending investigation results and follow-up visits. However, the case emphasises the challenges in differentiating ‘organic’, or ‘psychogenic’, paroxysmal movement disorders from FS.

In another single case (5%), a patient was referred to our service with a suspected FS diagnosis and reported multiple events of a single semiology characterised by a marked symptoms of dizziness and dissociation before the seizure, with subsequent collapse to the floor and reduced responsiveness. These had been previously labelled as functional in nature with a previous normal MRI and interictal routine EEG. VTEEG subsequently revealed that the patient had shown dual pathology of both FS and ES arising from the right temporal lobe. Before the FS, the patient reported a feeling of going in and out of their body before falling to the floor in a controlled way and remaining unresponsive and still for several minutes. During the ES, the patient reported a similar experience prior to their seizures before developing a loss of awareness, oral automatisms and fidgety movements of the right hand, all associated with epileptiform discharges. Interestingly, although distinctly different semiologies were witnessed to separate these events on VTEEG, the patient’s subjective experience of the two seizures was the same, leading them to believe they only had a single type of seizure.

Finally, one case (5%) of generalised epilepsy was misdiagnosed as suspected FS because seizures remained completely refractory to increasing doses of ASMs.

## Discussion

A diagnostic revision rate of 4.7% indicates that only a small minority of patients have their diagnosis revised from FS to ES, in comparison to the more common scenario of FS being misdiagnosed as ES.^4 6^ Furthermore, where diagnostic uncertainty between functional and epileptic events exists, most diagnostic revisions following VTEEG favour a conclusive FS diagnosis over an ES diagnosis.^11^ Our findings are in keeping with the diagnostic revision rate of 3.3% observed in another centre.^16^ This small proportion is additionally mirrored more broadly in studies of functional neurological disorders that track diagnostic revisions.^11 17^ However, our results highlight common misconceptions that may result in an FS misdiagnosis, and this small number of cases, therefore, serve an important educational purpose.

In frontal lobe and insular seizures, the absence of ictal EEG correlates does not preclude an epileptic diagnosis.^18^ However, it is possible that this phenomenon may have biased the referring clinician to classify the seizures as functional, especially, if it had not been possible to review videos of the seizures, or the videos had been reviewed by non-experts. Furthermore, because frontal lobe seizures often manifest with bizarre motor semiologies that may share common features with FS including bilateral movements with retained consciousness, these unconventional features may also bias the physician towards a functional diagnosis.^12 19^ These observations highlight the need for expert review of seizure videos when basing a diagnosis on recorded seizure semiology in the absence of EEG data. Caution should be exercised if events are observed to be short lived, stereotyped and nocturnal because these are strong indicators of frontal lobe epilepsy.^20^ Likewise, if a paroxysmal event associated with hyperkinetic movements looks atypical for an FS (eg, absence of pelvic thrusting, opisthotonos, eyelid closure, a fluctuating course and prolonged duration of motor features), or there is an absence of comorbid conditions or experiential symptoms before/during a seizure typically seen in FS, then, it is worth asking the question whether it might be a frontal lobe epileptic seizure.

Nocturnal seizures can present as a diagnostic challenge as demonstrated in this evaluation. On the one hand, nocturnal seizures may occur in focal epilepsies, especially, arising from the frontal lobe, generalised epilepsies and benign Rolandic epilepsy.^21^ As a result of the frequent lack of witnessed seizure descriptions, they are easily mistaken for parasomnias or FS.^22^ On the other hand, it is also common for patients with FS to report that their events arise from sleep despite VTEEG evidence of onset during wakefulness.^23^ The phenomenon of reported events arising from self-perceived sleep was highly suggestive of FS in one study, not being observed in ES.^24^ However, as illustrated by the findings in this case series, where individuals report frequent nocturnal events, it is important to consider VTEEG monitoring overnight because seizures that arise from EEG-defined sleep are invariably epileptic.^25^

Insular seizures often have overlapping features with seizures that arise in other cortical areas.^18^ Some of the clinical signs like those reported by our patient—choking, sweating, palpitations, hypersalivation and vomiting—can arise from insular seizures^26^ but may also be reported by patients with FS. Seizures arising from the posterior third of the insula may also evoke painful somatosensory phenomenon.^26^ Additionally, because the insula has a regulatory function over heart rate, seizures may coincide with bradycardia.^26^ Ictal bradycardia is uncommon compared with ictal tachycardia, also found to a lesser degree in FS.^27^ Although bradycardia can be associated with other seizure mimics,^28^ we have not found evidence that it occurs in FS.

Epilepsy is known to be a significant risk factor for the development of FS.^9^ There can therefore be diagnostic difficulty in patients with clear and obvious FS but also a historical but as yet unsubstantiated diagnosis of epilepsy. The consequences of ASM withdrawal in patients thought to suffer from FS but with a background of ES are reflected in published case reports. In one case, ASM reduction in a patient misdiagnosed as having FS precipitated worsening of frontal lobe seizures and caused post-ictal psychosis.^13^ Importantly, therefore, clinicians must consider the safety of withdrawing ASMs where an unsubstantiated, historical diagnosis of epilepsy exists, and the patient has a proven FS burden but no recent or current seizures with epileptic features. In these cases, it is possible that withdrawal of the ASMs will cause the re-emergence of genuine ES alongside the FS. At-risk groups for ES re-emergence in our study included patients with an absence of documented previous investigations either because they were not included in the referral documentation as a result of the diagnosis being made many years previously (eg, in childhood or as a young adult) or because the diagnosis of epilepsy was made abroad. In both cases, epilepsy had been diagnosed many years previously, but the patient had been free of all seizure types for a prolonged period of time before the onset of their FS. The cases reported here would therefore suggest there should be a low threshold for VTEEG in patient with FS with a historical diagnosis of epilepsy who wean their ASMs and then report the development of novel seizure types.

Brief ES with functional overlay or behavioural elaboration can obscure the diagnosis of epilepsy because the more obvious functional element may distract from the precipitating epileptic event, misleading the clinician.^29^ Functional elaboration may manifest with features found in our patients but also with phenomena of unresponsiveness and head rolling.^30 31^ Previous research has suggested that localised seizure activity may precipitate elaboration as described in a case series of three patients with focal seizures arising from the left or right hippocampi.^30^ Another study has implicated that elaboration may occur following right frontotemporal focal seizures and generalised absence seizures.^31^ Similarly, our research identified this phenomenon in seizures arising from either frontal lobe in addition to a generalised absence seizure. Elaborative movements during an ES may also be voluntary in focal seizures without impaired awareness arising from the supplementary motor area.^32^ This may too bias clinicians to suspect a functional cause.

The case of the misdiagnosed temporal lobe epilepsy seizure highlights the difficulty that can sometimes arise when basing a diagnosis solely on the description of peri-ictal symptoms. In both ES arising from the temporal lobe and FS, experiential symptoms such as dissociative phenomena including derealisation and depersonalisation can be present, although they are less common in ES.^33^ However, in ES, there may be other associated features characteristic of temporal lobe seizures such as manual or oral automatisms, which may aid differentiation and highlight the need for video or VTEEG in these cases.

It is unsurprising that clinicians feel the lack of ASM response may suggest a functional diagnosis. Evidence has suggested 6.4%–13% of patients with refractory epilepsy are misdiagnosed and suffer from FS.^1 34^ However, treatment of refractory epilepsy is common in clinic-based settings^35^ and our study suggests inadequate ASM response alone should not be used to revise an epileptic diagnosis. Indeed, given a proportion of refractory epileptics are incorrectly diagnosed as such because of inadequate ASM compliance and dosing,^36^ clinicians should primarily investigate these factors in the first instance.

Finally, we identified a patient with a working diagnosis of PKD, a disorder that may be misdiagnosed as functional or secondary to alternative neurological conditions such as epilepsy.^15 37^ PKD is a form of a paroxysmal movement disorder characterised by involuntary and transient uni/bilateral dystonic or choreiform movements that are precipitated by movement.^38^ PKD, alongside paroxysmal exercised-induced dyskinesia and paroxysmal non-kinesigenic dyskinesia (PNKD), are all paroxysmal dyskinesias and are well documented to be associated with genetic mutations.^38^ However, secondary causes have been described.^39^ Clinicians should be aware paroxysmal dyskinetic events occur without loss of consciousness and are precipitated by movement, such as standing from a seated position.^40^ Additionally, the presence of a positive family history, short event duration and significant response to carbamazepine is supportive of this diagnosis while clearly not specific, given these can be present in mimics such as epilepsy.^40^ An exception is PNKD, where attacks are often longer and are precipitated by alcohol, stress and coffee instead of movement.^38^ As with our patient, absence of ictal EEG changes may also suggest this diagnosis.^15^ However, as aforementioned, unusual hyperkinetic events may be secondary to frontal lobe epilepsy that may not have ictal correlates.

### Limitations

The rates of diagnostic error may be inflated due to the complexity of referred patients. Indeed, the complexity of the cohort referred to our centre was such that, although most patients had a provisional pre-admission diagnosis, there was still a need among referrers to confirm and reinforce that diagnosis. A group of patients with epileptic or provoked seizures with an autoimmune aetiology are conspicuous by their absence in this study. We rarely receive patients requiring intravenous immunoglobulin, plasma exchange or those with significant neuropsychiatric symptomatology. This may account for why this group have been identified as mimics in other clinical settings^14^ but not this study. The stringent admission criteria may result in missing additional mimics. In addition, because some pre-EEG impressions required a consensus decision, this exposed the study to interpretation bias.

Importantly, while this study does not contain a systematic assessment as to *why* cases were misdiagnosed, it does highlight *which* kinds of cases have the potential for misdiagnosis. In this regard, it is crucial for all clinicians diagnosing FS to rely not just on simple heuristics such as the absence of ictal, epileptiform EEG changes or presence of other comorbid functional neurological disorders but on the full panoply of evidence that may support a positive diagnosis of FS. These include seizure semiology, patient symptoms before and during a seizure, and comorbid psychiatric and medical diagnoses.

## Conclusion

We have identified those cases where the original diagnosis of FS has been revised to one of ES. These cases make up a very small absolute number, and typically, in those patients admitted to a VTEEG unit, their diagnosis remains unchanged or is revised in the opposite direction from ES to FS. Nevertheless, the identified cases highlight important learning points regarding the potential for the misdiagnosis of FS and the importance of expertise when analysing seizure recordings and accompanying electrophysiology. The mimics detected in this study include frontal lobe seizures where seizures can look bizarre and may not be associated with epileptogenic EEG changes, seizures arising from EEG-defined sleep, patients with both ES and FS, temporal lobe or insular seizures associated with marked experiential or autonomic symptoms respectively and paroxysmal movement disorders.

## Data Availability

All data relevant to the study are included in the article or uploaded as supplementary information.
